# Development of an e-learning system for teaching endoscopists how to diagnose early gastric cancer: basic principles for improving early detection

**DOI:** 10.1007/s10120-016-0680-7

**Published:** 2016-12-28

**Authors:** Kenshi Yao, Noriya Uedo, Manabu Muto, Hideki Ishikawa

**Affiliations:** 1 Department of Endoscopy, Fukuoka University Chikushi Hospital, 1-1-1 Zokumyouin, Chikushino, Fukuoka 818-8502 Japan; 2 Department of Gastrointestinal Oncology, Osaka Medical Center for Cancer and Cardiovascular Diseases, Osaka, Japan; 3 Department of Therapeutic Oncology, Graduate School of Medicine, Kyoto University, Kyoto, Japan; 4 Department of Molecular-Targeting Cancer Prevention, Graduate School of Medical Science, Kyoto Prefectural University of Medicine, Kyoto, Japan

**Keywords:** Endoscopic diagnosis, Gastric cancer, E-learning, International multicenter randomized controlled trial

## Abstract

We developed an internet e-learning system in order to improve the ability of endoscopists to diagnose gastric cancer at an early stage. The efficacy of this system at expanding knowledge and providing invaluable experience regarding the endoscopic detection of early gastric cancer was demonstrated through an international multicenter randomized controlled trial. However, the contents of the system have not yet been fully described in the literature. Accordingly, we herein introduce the contents and their principles, which comprise three main subjects: technique, knowledge, and experience. Since all the e-learning contents and principles are based on conventional white-light endoscopy alone, which is commonly available throughout the world, they should provide a good reference point for any endoscopist who wishes to devise learning materials and guidelines for improving their own clinical practice.

## Introduction

In many countries, gastric cancer is not diagnosed until it is at an advanced stage. Advanced endoscopic imaging such as magnifying endoscopy with narrow-band imaging is generally used in Japan [1] and some Western countries [2], but is still rarely used in developing countries, where the prevalence of gastric cancer is high. Conventional white-light endoscopy is a standard endoscopy technique throughout the world. However, to date, there has been no standardized learning system based on conventional white-light endoscopy which could be commonly applied to screening endoscopy for the detection of early gastric cancer.

We constructed an e-learning system and finalized a global study to test its usefulness through a randomized controlled trial [3]. In that trial, after receiving a pre-test, participants were randomly allocated to either an e-learning or a non-e-learning group. Only those in the e-learning group gained access to the e-learning system. Two months after the pre-test, both groups received a post-test. Five hundred fifteen endoscopists from 35 countries were assessed for eligibility, and 332 were enrolled in the study, with 166 allocated to each group. Of these, 151 participants in the e-learning group and 144 in the non-e-learning group were included in the analysis. The mean improvement rate (standard deviation) in the e-learning and non-e-learning groups was 1.24 (0.26) and 1.00 (0.16), respectively (*P* < 0001, *t* test). We have already reported that the e-learning system was useful for improving the endoscopic detection of early gastric cancer, irrespective of pre-test score, endoscopist experience, or geographical area [3]. However, to date, the contents of this e-learning system have not been fully described in the literature.

Accordingly, the aim of this article is to demonstrate the contents of the e-learning system so that medical practitioners can obtain information regarding what they need to learn and how they should teach endoscopy practice in order to improve the detection of early gastric cancer using white-light endoscopy alone.

## E-learning contents

In order to improve the endoscopic detection of early gastric cancer, doctors need to acquire basic principles that comprise the three keys to detection: technique, knowledge, and experience [4, 5]. Accordingly, the e-learning contents were devised as follows [3]:

1. Technique
  Lecture (video clips and slides).
    1. Ideal preparation with mucolytic and defoaming agents.
    2. Use of anticholinergic agents.
    3. Avoiding blind spots:
      Gastric wall should be distended by air insufflation.
      Mucus and bubbles should be rinsed off using irrigating water with defoaming agent.
      Entire stomach should be mapped: systematic screening of the stomach (SSS).
2. Knowledge
  Test (ten questions without answers).
  Lecture (video clips and slides).
    1. General: normal appearance and chronic gastritis.
    2. Detection: awareness of signs of suspicious lesions.
    3. Awareness of multiple synchronous lesions.
    4. Characterization using the GUP system: G gastritis-like lesions, U ulcerative lesions, and P polypoid lesions.
      Ten (ten questions with answers and descriptions).
3. Experience: 100 cases for EGC detection training
  Mock test (ten cases with scores and no answers).
  Random version of the 100 cases.
  Systematic version of the 100 cases.
  Random version of the 100 cases.
  Mock test (ten cases with scores and answers).

### Technique

Video clips and slides were uploaded in order to explain the requisite endoscopic technique for improving the detection of early gastric cancer.

#### Ideal preparation with mucolytic and defoaming agents

The correct preparation for endoscopic examination is mandatory to minimize time and effort during the procedure, i.e., the removal of mucus and froth from the mucosal surface. Thirty minutes before the procedure, patients are asked to drink a mixture of water with mucolytic and defoaming agents. The formula in Japan is 100 mL of water with 20,000 U Pronase® (Kaken Pharmaceutical, Tokyo, Japan), 1 g of sodium bicarbonate, and 10 mL of dimethylpolysiloxane (20 mg/mL, Horii Pharmaceutical Ind., Osaka, Japan). However, Pronase® is not available for use in all countries. An alternative mixture comprises 100 mL of water mixed with 2 mL of acetylcysteine (200 mg/mL Parvolex®, Celltech, Slough, UK; or Mucomyst, Bristol-Myers Squibb, New York, NY, USA), and 0.5 mL activated dimethicone (40 mg/mL, Infacol®, Forest Laboratories, Dartford, UK).

#### Use of anticholinergic agents

In the normal physiological state, the gastric wall is always moving due to peristalsis. In order to detect subtle mucosal changes, we need to carefully scan the entire mucosal surface. However, peristalsis makes it difficult for the endoscopist to obtain static observations. Accordingly, intravenous or intramuscular administration of an anticholinergic agent such as 10–20 mg of scopolamine butylbromide (Buscopan®) is recommended just before insertion of the endoscope, even for upper gastrointestinal (GI) screening endoscopies. If there are contraindications to the use of anticholinergic agents (e.g., cardiovascular disease), 1 mg of glucagon can be administered to inhibit peristalsis.

#### Avoiding blind spots

During the endoscopy, in order to avoid blind spots, a standardized approach to map the entire stomach needs to be employed. A basic technique for avoiding blind spots involves the following procedures: (1) distending the gastric wall by air insufflation, (2) rinsing mucus and froth off the gastric mucosa by irrigating with water and a defoaming agent, and (3) mapping the entire stomach. The need for these steps is explained below.

1. If the gastric wall is not fully distended, we may fail to detect a cancer on the greater curvature, even a rather advanced cancer. The lecture demonstrates a very good example of advanced gastric cancer on the greater curvature of the lower gastric body. With insufflation of a small amount of air, the appearance is normal (Fig. 1a). However, when more air is insufflated and the gastric wall together with the greater curvature with gastric folds is distended, a distinct lesion, suggestive of advanced gastric cancer, becomes evident (Fig. 1b).

**Fig. 1a–b:**
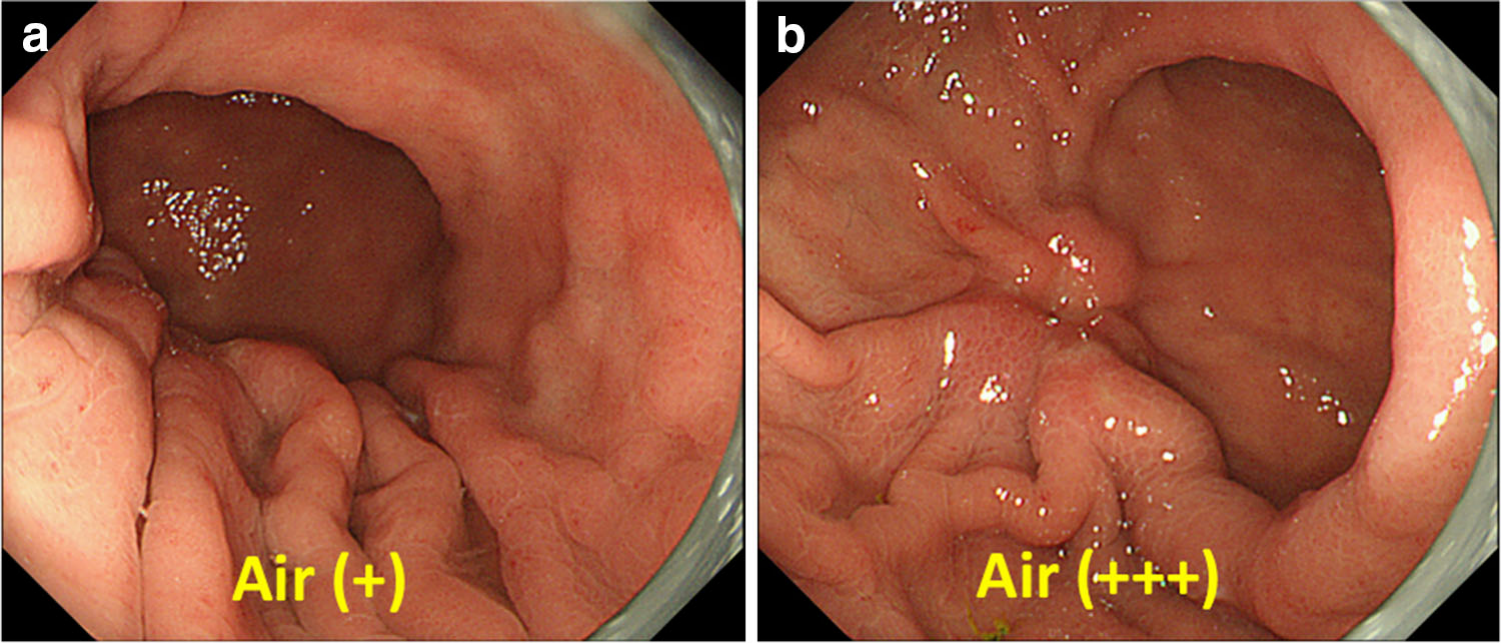
Conventional white-light endoscopic findings of an advanced gastric cancer located at the greater curvature. **a** With insufflation of a small amount of air, the appearance is normal. **b** With insufflation of a large amount of air, a distinct advanced cancer becomes evident
2. A short video clip of an endoscopy examination is included in the video lecture in order to demonstrate why the mucus and froth need to be rinsed off. Even after patients drink a mixture of water with mucolytic and defoaming agents, cases with copious mucus and froth within the stomach are sometimes encountered (Fig. 2a). However, when the mucus and froth are rinsed off the gastric mucosal surface through irrigation with water and a defoaming agent, even subtle lesions can be detected (Fig. 2b).

**Fig. 2a–b:**
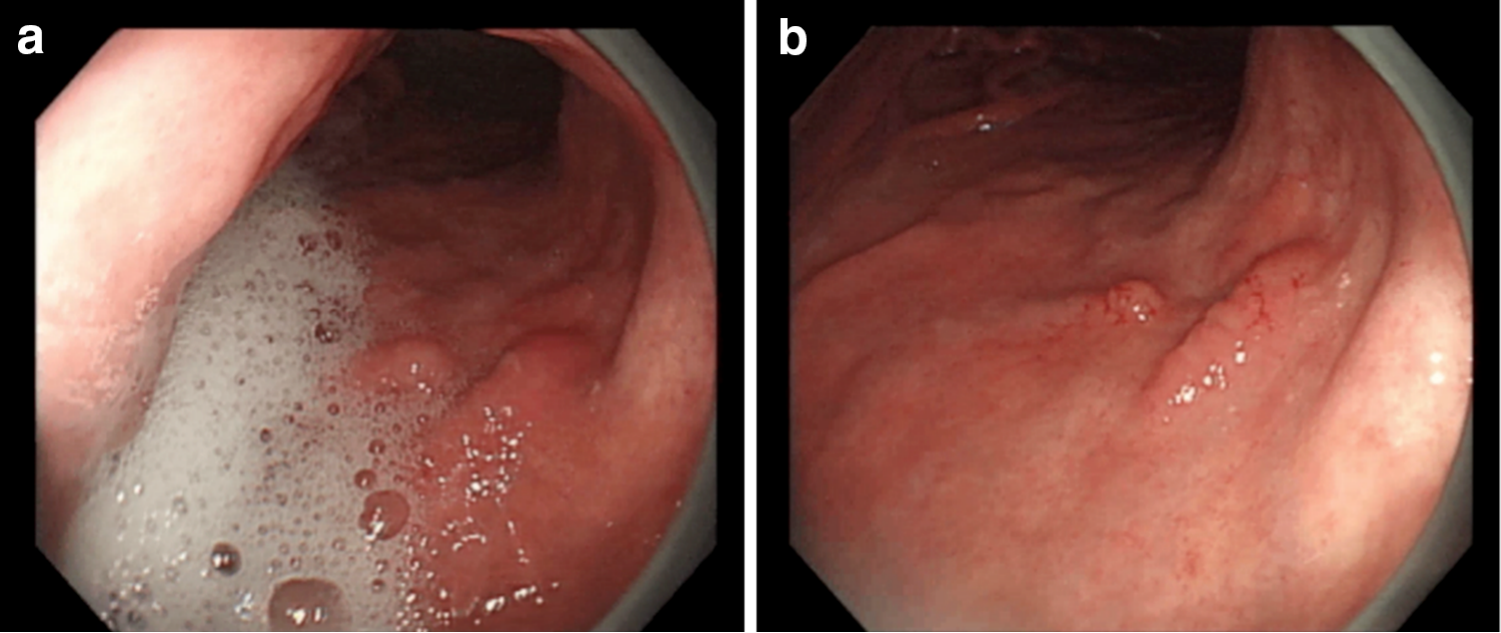
A video clip which demonstrates the need to rinse mucus and froth off the mucosal surface during endoscopic examination. **a** When copious mucus and froth are present, we are unable to detect subtle lesions. **b** After the mucus and froth have been rinsed off the gastric mucosal surface by irrigating with water and a defoaming agent, we are able to detect a subtle lesion of early gastric cancer
3. Within the e-learning contents, the “systematic screening protocol for the stomach (SSS)” is introduced in order to help map the entire stomach (Fig. 3) [4, 5]. In the SSS, pictures are arranged according to the order of the procedure, and pictures of three- or four-quadrant views can be taken in either a clockwise or counterclockwise manner. Overall, the SSS series comprises 22 endoscopic photos. The SSS is a basic concept which shows the minimum required standard. If an endoscopist finds lesions, additional pictures can be taken. One of the good features of the SSS is that it is easy to remember. In addition, the amount of air increases in accordance with the order in the protocol. This means that it is efficient. If the endoscopy system does not connect to an image-filing database system, the SSS should be used as check points.

**Fig. 3:**
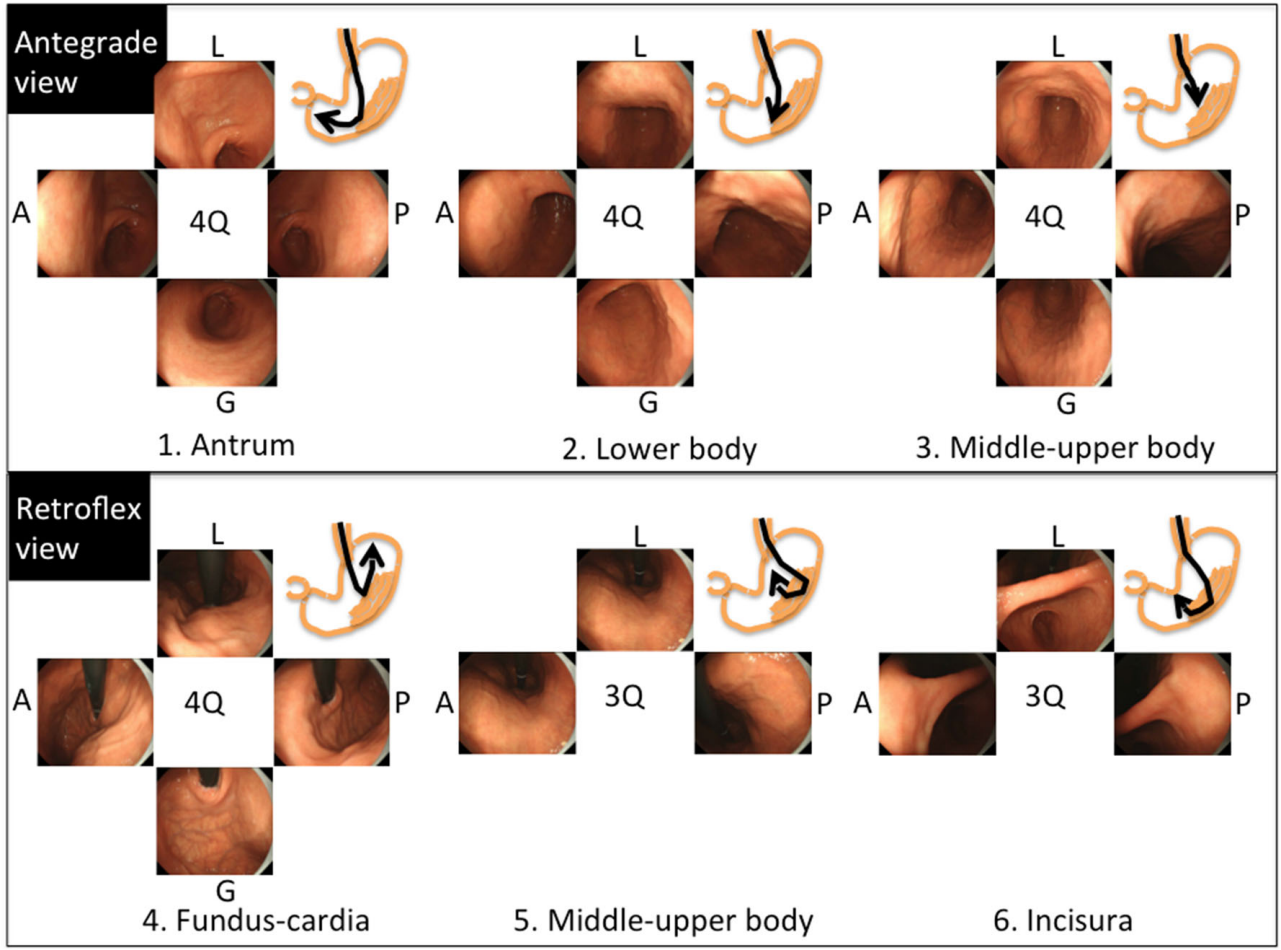
Systematic screening protocol for the stomach (SSS). The SSS should be initiated as soon as the scope is inserted into the gastric antrum. In the antegrade view, endoscopic images of four quadrants of the gastric antrum, body, and *middle*–*upper* body are obtained and then, in the retroflex view, images of the four quadrants of the gastric fundus and cardia, and three quadrants of the gastric *middle*–*upper* body and incisura are taken. Overall, the SSS comprises 22 endoscopic images. *A* anterior wall, *G* greater curvature, *L* lesser curvature, *P* posterior wall, *Q* quadrant

### Knowledge

In the lecture regarding knowledge, emphasis is placed on the following subjects: (1) the endoscopic appearance of normal gastric mucosa versus that of an abnormal high-risk condition for gastric cancer, such as atrophic gastritis or intestinal metaplasia; (2) how to detect suspicious lesions in the stomach; and (3) how to characterize a detected lesion according to macroscopic type, i.e., gastritis-like (G), ulcerative (U), and polypoid (P) lesions. This diagnostic system was named the GUP system and was first proposed specifically for use in this e-learning system.

#### Normal mucosa versus chronic atrophic gastritis

**Fig. 4a–b:**
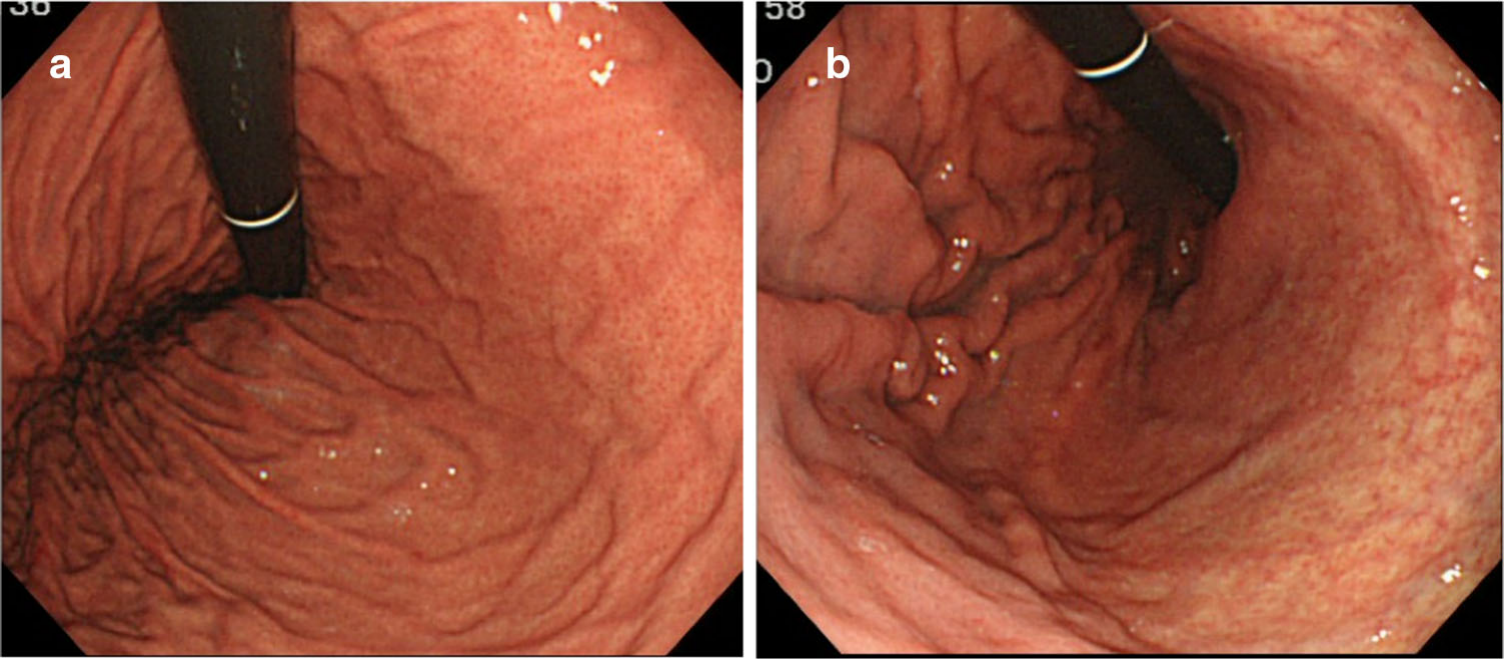
Conventional white-light endoscopic findings for the gastric body. **a** Normal gastric mucosa without HP infection. **b** Gastric mucosa with chronic atrophic gastritis associated with HP infection. Visible vessels and an absence of folds in the gastric body mucosa are characteristic of chronic atrophic gastritis

**Fig. 5:**
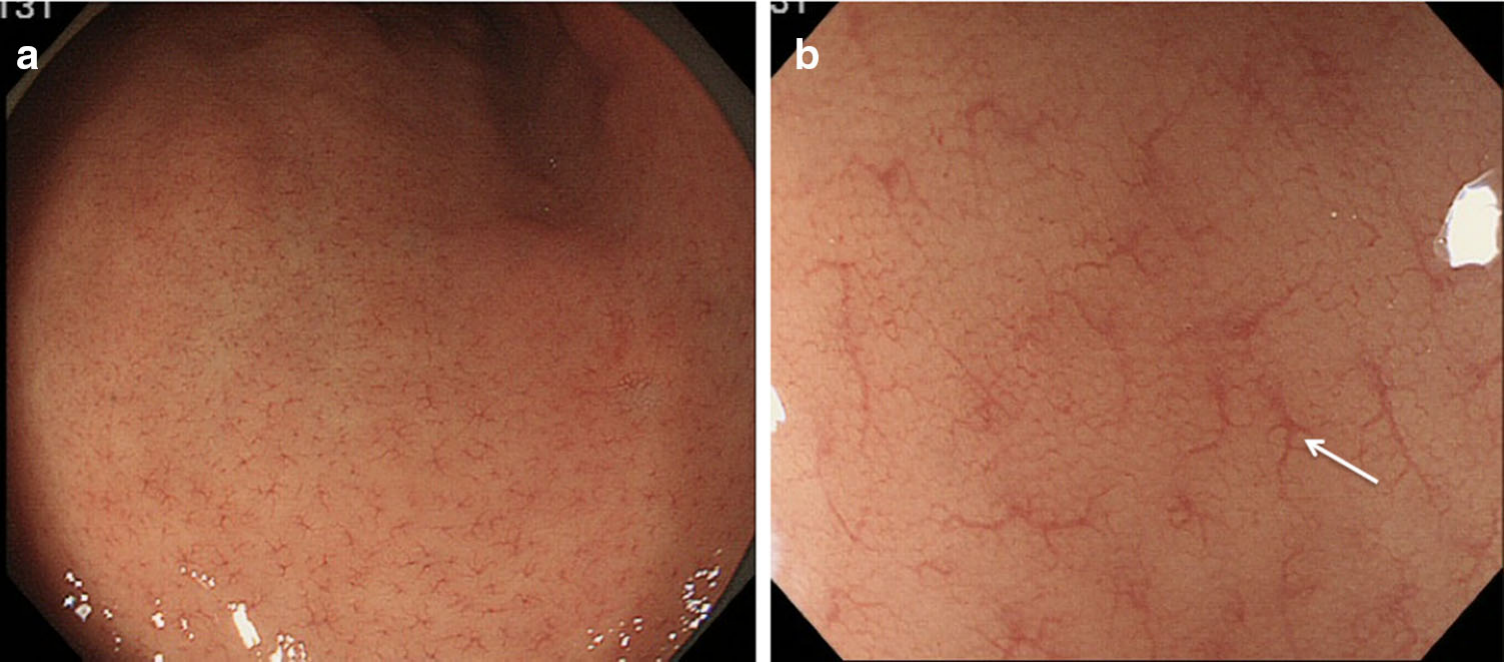
**a** Conventional white-light endoscopic findings for the gastric body. Regular arrangement of collecting (RAC) venules is present in normal mucosa. **b** Magnifying white-light endoscopic findings for the gastric body. The small vascular-spider-like vessels in** a** correspond to regularly arranged venules (arrow)

As soon as the scope is inserted into the stomach, the endoscopist needs to determine, using endoscopic inspection alone, whether risk factors for gastric cancer are present in the background mucosa (such as *Helicobacter pylori* (HP)-associated gastritis, gastric atrophy, or intestinal metaplasia). If the appearance of the gastric mucosa is normal (Fig. 4a) then lesions suspicious for gastric cancer are less likely. Figure 4b demonstrates how visible vessels and the absence of folds in the gastric body mucosa are characteristic of chronic atrophic gastritis. A unique sign called RAC (regular arrangement of collecting venules) is introduced [6]. By careful endoscopic observation of the normal gastric body mucosa, vascular spider-like small vessels can be discerned (Fig. 5a). Observation of the microvascular architecture using magnifying endoscopy reveals that these small vessels correspond to collecting venules (Fig. 5b). If these collecting venules are arranged regularly, the sensitivity and the specificity for diagnosing normal gastric body mucosa without HP infection are 93.8 and 96.2%, respectively [6].

#### How to detect and characterize suspicious lesions in the stomach

The diagnostic process can be divided into two steps: detection and characterization. Detection requires good endoscopic technique and thorough knowledge. The lecture describes the procedure for improving the detection and characterization of early gastric cancers.

##### Detection

In order to detect early gastric cancer, endoscopists need to be aware of the signs of suspicious lesions. The Paris classification of macroscopic appearance was proposed in 2003 with reference to Japanese systems of classification [7]. The Paris classification is now commonly used worldwide. However, this classification is not very practical in a clinical setting when detecting and characterizing suspicious lesions at screening endoscopy. Accordingly, the diagnostic system was modified to make it more suitable for adoption by endoscopists in their own clinical practice. Briefly, the visible endoscopic findings that endoscopists need to be concerned about can be divided into just three types: gastritis-like lesions [G: gastritis vs superficial cancer (0 IIa, IIb, IIc)], ulcerative lesions [U: peptic ulcer vs superficial cancer with ulceration (0 III)], and polypoid lesions [P: polyp vs polypoid cancer (0 I)]. Based on this concept, which seems to be readily endorsed worldwide, endoscopists attempt to detect the presence of suspicious lesions. Provisionally, this diagnostic system was named “the GUP system” [3]. This is an original, brand new idea, developed specifically for this e-learning project. Instructions based on this GUP system are given throughout the e-learning course.

For the purpose of detection, the first thing that needs to be mastered is how to be aware of the presence of suspicious lesions. The most common markers for detection are (1) surface change and (2) color change. Other markers, namely abrupt changes in the vascular/mucosal pattern, changes in light reflection, and spontaneous bleeding, are also of importance. Early gastric cancers of both the polypoid and ulcerative types are easily detected by surface change alone, provided that the endoscopist follows the SSS protocol with optimum preparation. On the other hand, superficial mucosal lesions that mimic gastritis—that is, gastritis-like lesions (G)—are very difficult to detect, even with optimum preparation and the best technique. Accordingly, endoscopists need to be particularly aware of the key signs for detecting superficial gastritis-like neoplasia.

**Fig. 6a–b:**
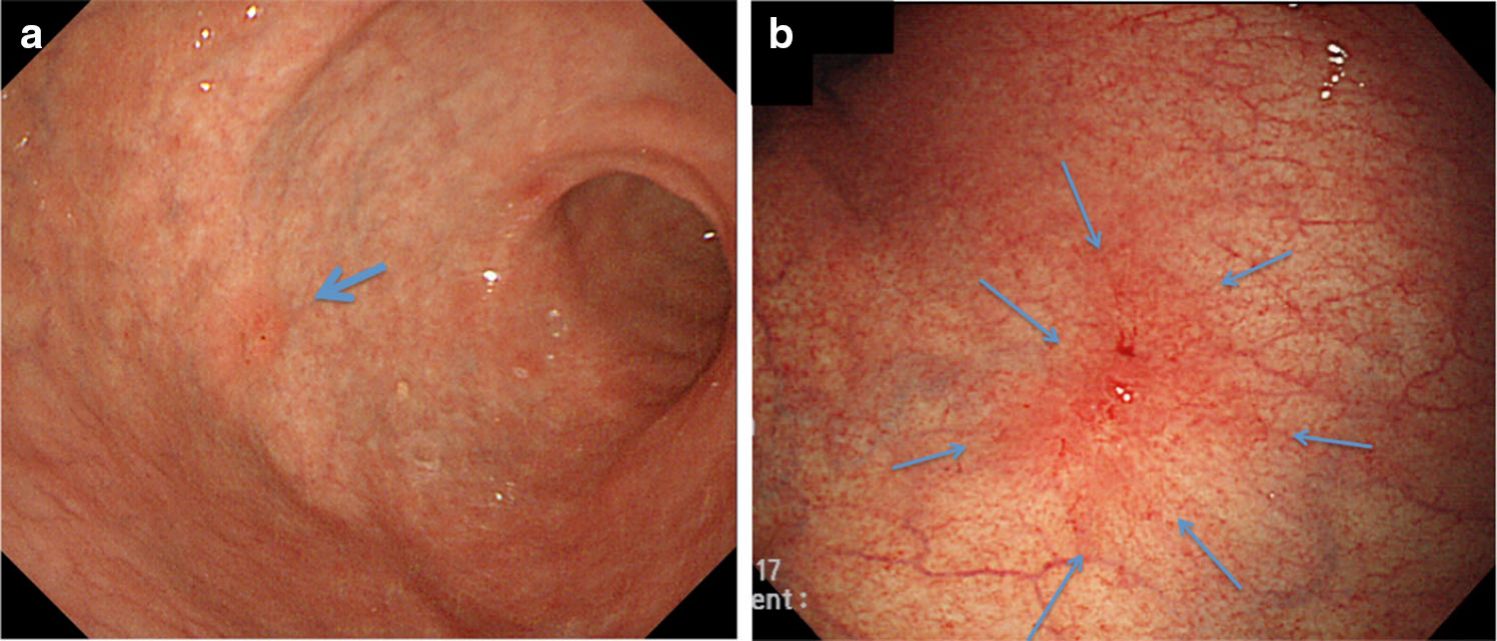
Examples of endoscopic markers for detection by conventional white-light endoscopy. **a** Marker, surface change: a very small early gastric cancer of the superficial depressed type (gastritis-like lesion) as indicated by an *arrow* in the gastric antrum. **b** Marker, color change: a superficial depressed type which mimics gastritis (gastritis-like lesion) in the gastric body. *Arrows* show a well-demarcated reddened lesion with abrupt interruption of the vascular pattern in the background mucosa

The first key marker is surface change. Figure 6a shows an endoscopic photo of a very small early gastric cancer of the superficial depressed type. Since this lesion is very small and only shows subtle changes, with morphology mimicking an erosive gastritis lesion, it is very difficult to detect this lesion. However, it is essential to be aware that such subtle surface changes can be important signs of early gastric cancer. The second important marker is color change. A well-demarcated area with color change is a useful indicator of a gastritis-like cancer. Figure 6b presents the endoscopic findings of superficial depressed type which mimics gastritis (gastritis-like lesion). In the background atrophic mucosa, a clear vascular pattern located in the deeper part of the mucosa and submucosa can be visualized. If the background vascular pattern is traced, it soon becomes apparent that there is a well-demarcated area where the vascular pattern is abruptly interrupted at the margin of the lesion. This is indeed a useful finding for early detection.

##### Characterization

**Fig. 7a–b:**
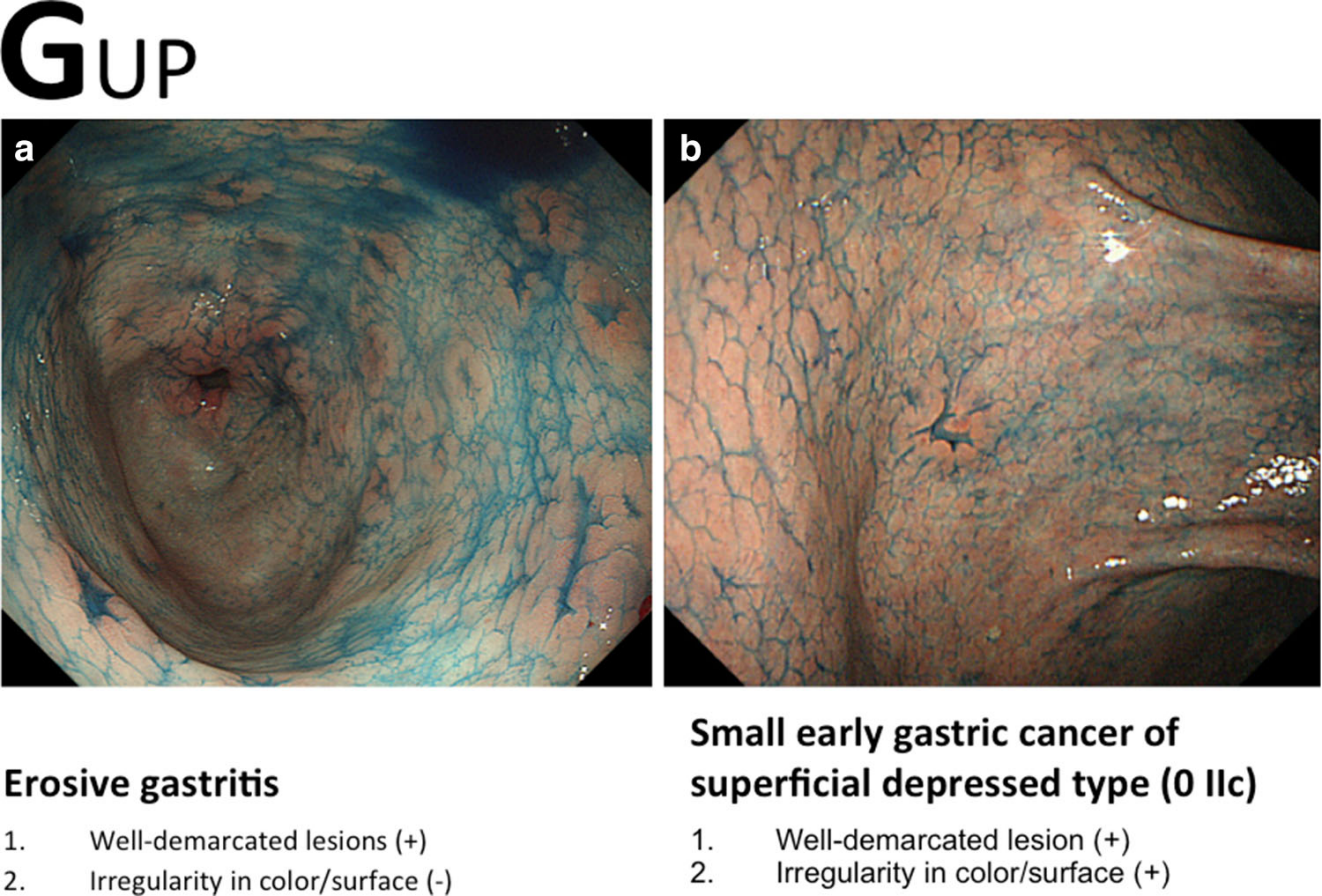
Examples of the GUP system and its criteria for characterization by conventional white-light endoscopy with chromoendoscopy (gastritis-like lesion). **a** Erosive gastritis. **b** Small early gastric cancer of superficial depressed type (0 IIc)

For this e-learning system, considerable effort was directed into creating very simple criteria for making a diagnosis based on conventional white-light endoscopic appearance alone. For characterization, two distinct markers, namely color and surface morphology, should be applied to the interpretation of conventional endoscopic findings. Chromoendoscopy using indigo carmine is useful for enhancing the surface pattern (Fig. 7). The differential diagnosis is made using the following criteria [4, 8]:

1. Well-demarcated border
2. Irregularity in color/surface pattern

**Fig. 8a–b:**
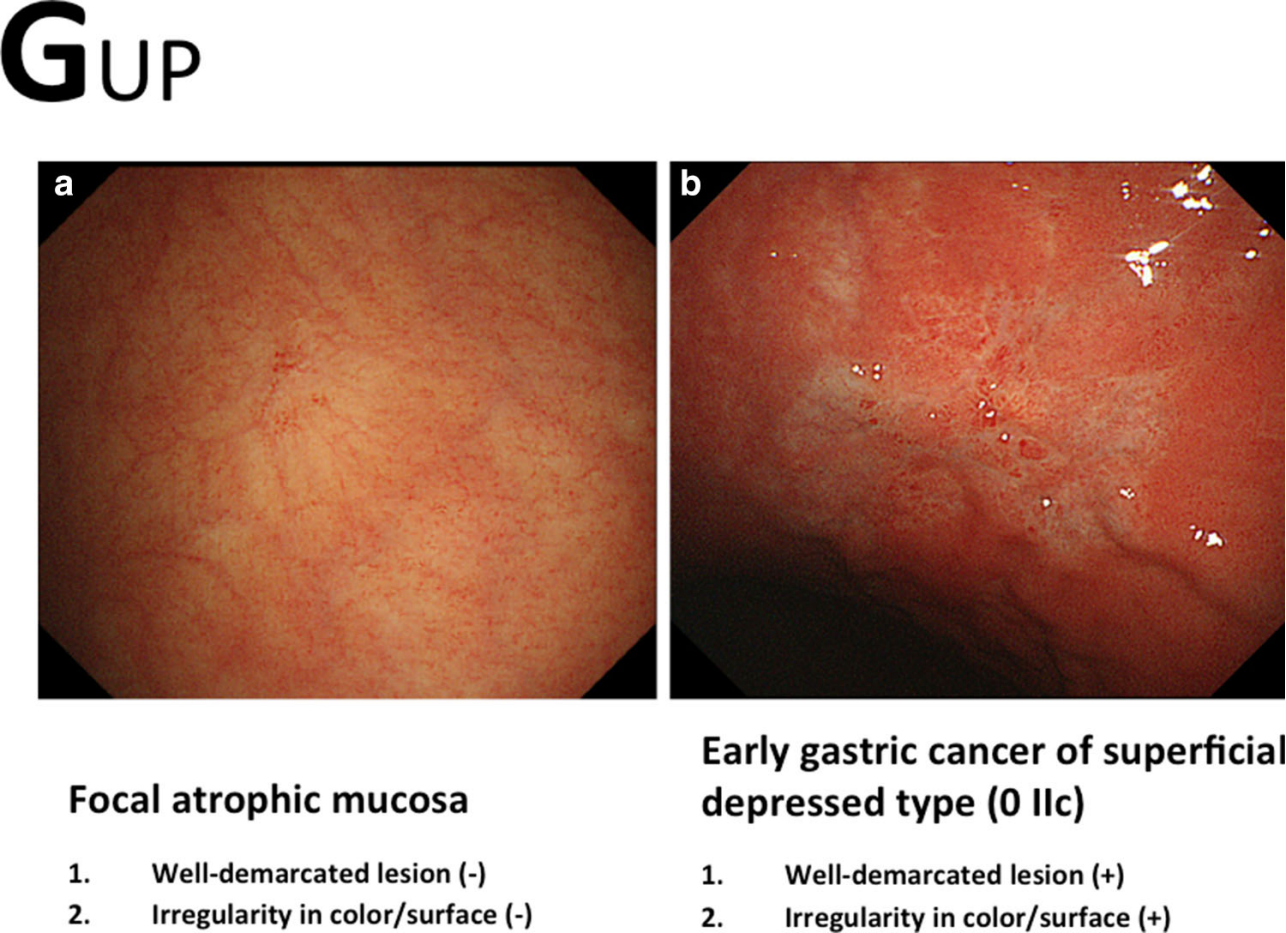
Examples of the GUP system and its criteria for characterization by conventional white-light endoscopy (gastritis-like lesion). **a** Focal atrophic mucosa. **b** Early gastric cancer of superficial depressed type (0 IIc)

**Fig. 9a–d:**
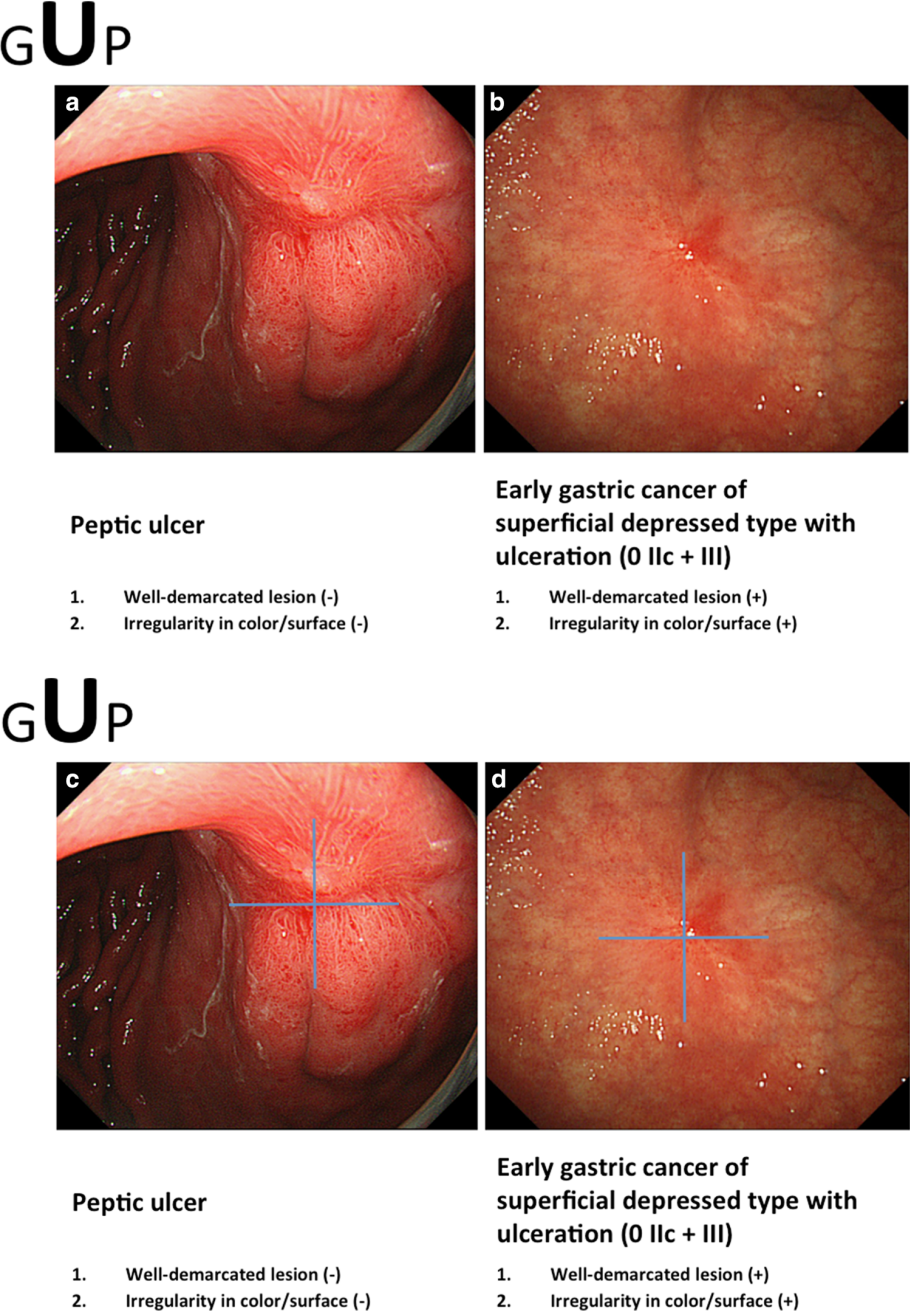
Examples of the GUP system and its criteria for characterization by conventional white-light endoscopy (ulcerative lesion). **a** Peptic ulcer, scar. **b** Early gastric cancer of superficial depressed type with ulceration (0 IIc + III). **c** Symmetrical distribution of the color texture is evident when using a magic cross. **d** Asymmetric distribution of the redness is easily recognized using a magic cross

**Fig. 10a–d:**
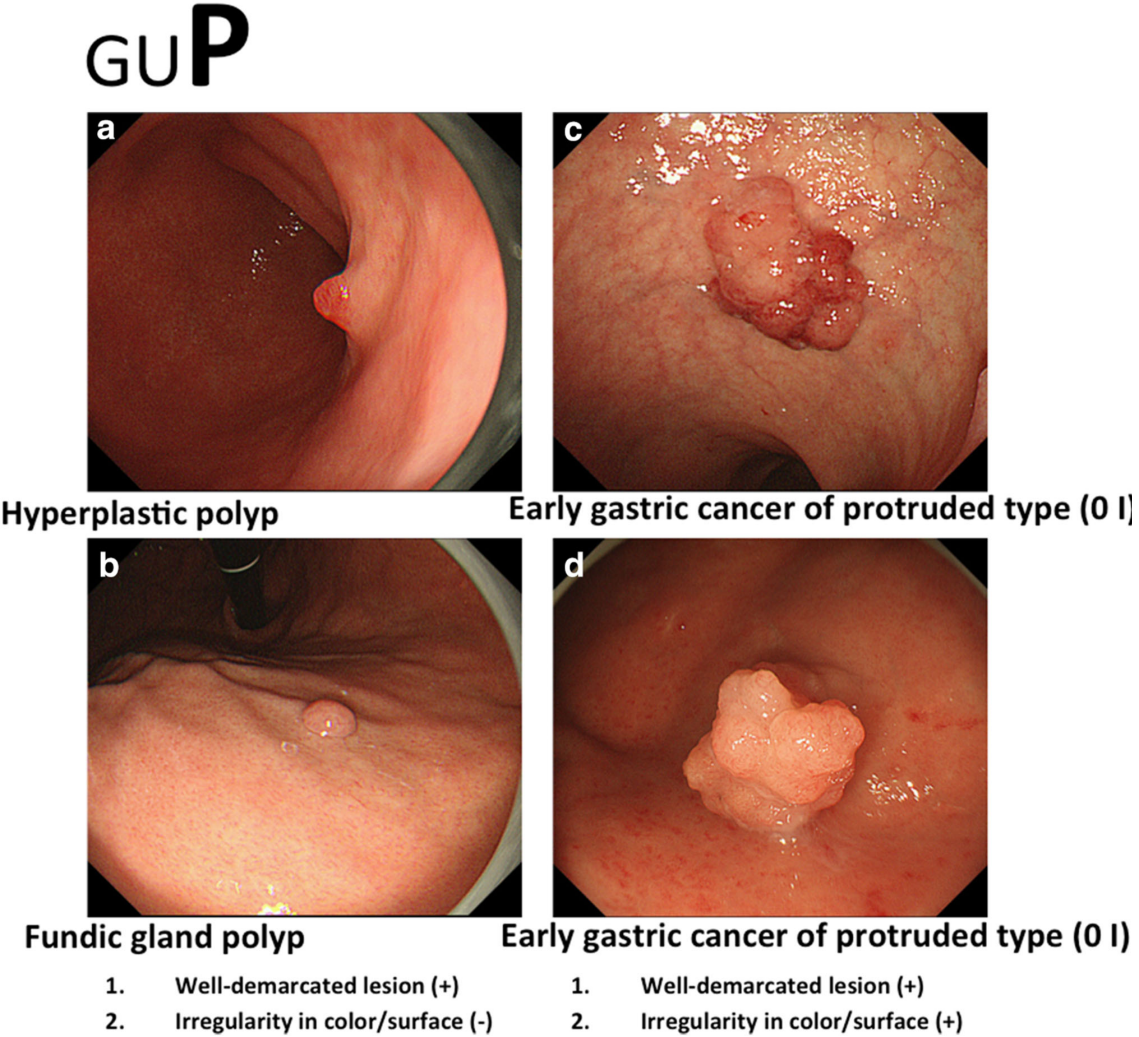
Examples of the GUP system and its criteria for characterization by conventional white-light endoscopy (polypoid lesion). **a** Hyperplastic polyp 1. **b** Fundic gland polyp. **c** Early gastric cancer of protruded type (0 I). **d** Early gastric cancer of protruded type (0 I)

If conventional white-light endoscopy and/or chromoendoscopy findings fulfill both criteria, an endoscopic diagnosis of early gastric cancer is made; if not, the diagnosis is non-cancer (Figs. 8, 9, 10). In this e-learning system, these criteria are useful and acceptable as they help overseas doctors to correctly diagnose early gastric cancer.

### Experience

After acquiring the technique and necessary knowledge, the endoscopist needs to obtain substantial experience in order to make a differential diagnosis of cancer and non-cancer. In fact, experienced endoscopists in Japan make a diagnosis at a glance immediately after detecting the cancer. However, increasing the experience of endoscopists in other countries is a major stumbling block, since they lack opportunities to obtain the necessary experience within their own clinical practice. In order to compensate for this lack of experience, an original self-exercise test program, the so-called “100 cases for EGC (early gastric cancer) detection training,” is provided as part of this e-learning system.

**Fig. 11a–c:**
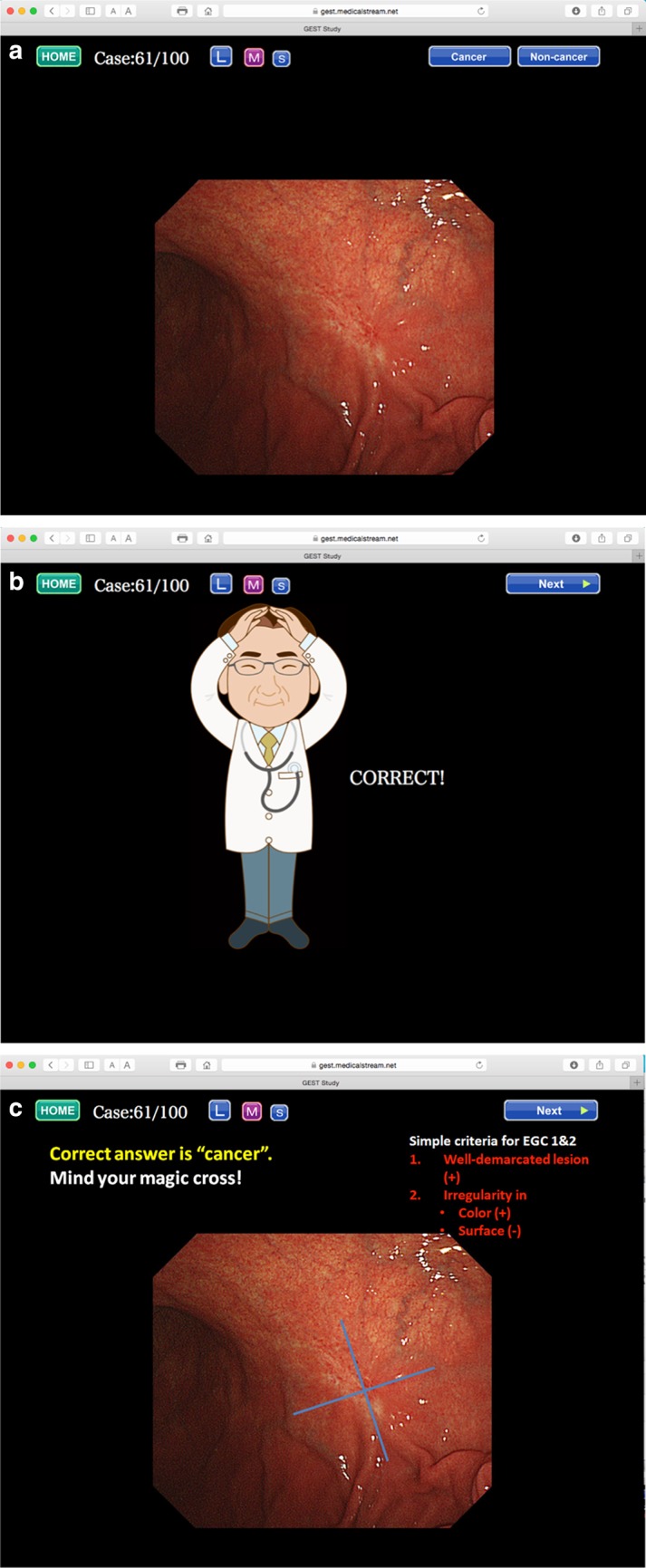
An example from the 100 cases for EGC detection training. **a** First slide, **b** second slide, **c** third slide

The system includes 100 cases which comprise 50 early gastric cancers and 50 non-cancerous lesions. We prepared 100 sets of images, with each set comprising three slides dealing with one case (Fig. 11). The first slide shows one endoscopic photo where one lesion is shown. First, the participant should choose and click whether or not the lesion is cancer (Fig. 11a). Immediately after pressing the cancer/non-cancer button, the illustration in the second slide reveals the answer to be correct/incorrect (Fig. 11b). In the third slide, there are brief instructions on how to characterize the endoscopic findings in order to make a correct diagnosis, with a review of the original endoscopic findings in that particular case (Fig. 11c). The cases are arranged either in random order or systematic order according to the GUP system. Repetition of a quick question followed by a quick answer for 100 cases offers the participants substantial experience in discerning between cancer and non-cancer in their own minds. Furthermore, mock tests are prepared before and after the 100 cases for EGC detection training so that participants can check their progress. Participants can repeat the training and mock tests as often as they wish until they feel sufficiently confident.

## Discussion

Until now, there was no literature presenting standardized learning contents that were demonstrated as useful for enabling endoscopists worldwide to improve their endoscopic detection of early gastric cancer based on conventional white-light endoscopy alone. We have summarized the contents in this article according to (1) technique, (2) knowledge, and (3) experience. For those three subjects, endoscopists can acquire knowledge and technique by attending conventional lectures or hands-on seminars. However, it is difficult for learners to accumulate experience in a single lecture or hands-on seminar. Therefore, we incorporated “100 cases for EGC detection training” into this e-learning system. We believe that simple but repetitive practice is useful for maintaining ability in any learning opportunity [9]. The concepts included in this learning system can be generalized as learning contents that would be suitable not only for other gastrointestinal organs (esophagus, large intestine, etc.) but also for the general medical field. An e-learning system based on the internet offers a huge advantage over conventional teaching methods, such as one-to-one tutorial teaching, hands-on seminars, lectures, and printed literature, since there is no limit to the number of learners who can participate.

As we described previously [3], the limitation of the study was that the outcome measurement was assessed not by the detection rate in clinical practice but by e-test scores. However, we are now collecting data on the clinical detection rate of early gastric cancer from the participants, and we intend to compare the early detection rate obtained after a participant experienced e-learning with that obtained prior to e-learning (UMIN: R000012039). Furthermore, we are organizing “train the trainer” (TTT) courses in countries where the incidence of gastric cancer is high. In this course, we are training a certain number of candidate trainers using the same contents, while at the same time we are providing hands-on instruction in clinical practice. In the near future, the early detection rate in actual clinical practice before and after the TTT course will become clear. In addition, we have started a global multi-center trial of EGC screening through a teleconference system using the learning contents described in this article [JSPS Core-to-Core Program (B. Asia-Africa Science Platforms)]. Another limitation was that the opportunity to partake in this e-learning course was limited to participants who were registered with this trial. However, we are pursuing the possibility of opening up this e-learning system using an official internet website as a platform so that any endoscopist in any country can access this system freely.

In conclusion, good practice based on good technique, knowledge, and adequate experience improves the ability of a doctor to detect gastric cancer or other cancers at an early stage. The aim of introducing the contents of this e-learning system in this article is to enable any endoscopist in any country to benefit from such practice. This e-learning system is likely to contribute to improved human health and welfare by reducing the mortality from gastrointestinal cancer.
